# Virtual patients to explore and develop clinical case summary statement skills amongst Japanese resident physicians: a mixed methods study

**DOI:** 10.1186/s12909-016-0571-y

**Published:** 2016-02-01

**Authors:** Brian S. Heist, Naoki Kishida, Gautam Deshpande, Sugihiro Hamaguchi, Hiroyuki Kobayashi

**Affiliations:** University of Pittsburgh School of Medicine, Pittsburgh, PA USA; Adjunct Teaching Faculty, Teine Keijinkai Hospital, Sapporo, Japan; Department of General Internal Medicine and Infectious Diseases at Teine Keijinkai Hospital, Sapporo, Japan; St. Luke’s International Hospital, Tokyo, Japan; Department of Internal Medicine, University of Hawaii, Honolulu, HI USA; Hokkaido General Internal Medicine Education Center, Ebetsu, Japan; Mito Medical Center, Tsukuba University Hospital, Mito, Japan; University of Pittsburgh School of Medicine, Division of General Internal Medicine, UPMC Shadyside Hospital - North Tower #306, Pittsburgh, PA 15232 USA

**Keywords:** Clinical reasoning, Summary statement, Post-graduate, Medical education, Virtual patient, Japan

## Abstract

**Background:**

In Western clinical training, formulation of a summary statement (SS) is a core exercise for articulation, evaluation, and improvement of clinical reasoning (CR). In Japanese clinical training, structured guidance in developing CR, including opportunity for SS practice, is uncommon, and the present status of case summarization skills is unclear. We used Virtual Patients (VPs) to explore Japanese junior residents’ SS styles and the effectiveness of VPs on improving SS quality.

**Methods:**

All first-year junior resident physicians at 4 residency programs (*n* = 54) were assigned randomized sequences of 5 VP modules, rolled out at 6 day intervals. During each module, participants free-texted a case summary and then reviewed a model summary. Thematic analysis was used to identify SS styles and each SS was categorized accordingly. Frequency of SS styles, and SS CR quality determined by 1) an internally developed Key Feature rubric and 2) demonstration of semantic qualification, were compared across modules.

**Results:**

Four SS styles were identified: numbered features matched to differential diagnoses, differential diagnoses with supportive comments, feature listing, and narrative summarization. From module #1 to #5, significant increases in the narrative summarization SS style (*p* = 0.016), SS CR quality score (*p* = 0.021) and percentage of semantically driven SS (*p* = 0.003) were observed.

**Conclusions:**

Our study of Japanese junior residents identified distinct clinical case summary statement styles, and observed adoption of the narrative summarization style and improvement in the CR quality of summary statements during a series of VP cases.

**Electronic supplementary material:**

The online version of this article (doi:10.1186/s12909-016-0571-y) contains supplementary material, which is available to authorized users.

## Background

Japanese medical training priorities are influenced by a traditional passive apprenticeship model and Japan’s 19^th^ century adoption of the German medical education system [1, 2]. Financial and cultural barriers continue to delay reform [1–4]. One result of this history has been limited teaching of fundamental skills including clinical reasoning [2, 3, 5]. There is also substantial variation in the training experience of young Japanese physicians in terms of patient encounters and educational time [6]. Recently, in a pragmatic response to improve this situation, Japan has seen the emergence of widely available paper-based publications attempting to teach clinical reasoning to Japanese physician trainees [7].

In Western medical training, education in clinical reasoning has traditionally involved case-based discussions and case presentations [8–11]. With the development of computer-assisted instruction and high-speed internet access, these activities have been increasingly supplemented by computer-based “virtual” patient (VP) cases [12]. While not yet widespread in Japan, enthusiasm toward VPs as an educational modality has been previously demonstrated among Japanese physician trainees [7].

In the context of case discussion and VPs, formulation of a summary statement is a core exercise for the articulation, evaluation, and in turn, the improvement of CR [9, 11, 13, 14]. There are two specific educational benefits associated with summary statement practice. First is the improvement in problem representation, the characterization of key features of the case using semantic qualifiers. Semantic qualifiers are abstract descriptors (e.g. acute versus gradual onset, intermittent versus constant occurrence) that help distinguish potential diagnoses. Analysis of summary statements has demonstrated classifiable differences in semantic content that correlate with diagnostic accuracy and quality of reasoning [15, 16]. Second, summary statements help develop illness scripts [17]. Illness scripts are conceptual models of clinical conditions, including enabling conditions and clinical consequences (i.e. history of present illness and exam findings) and how their variations fit together [18, 19]. With feedback from supervising physicians to guide problem representation and illness script development, the physician trainee encapsulates and integrates clinical experiences into an organized network of knowledge that can be effectively applied to future clinical practice [8, 19, 20].

Structured guidance from clinician educators, including daily opportunity for case discussion and summary statement practice, is uncommon within Japanese medical education [3, 6], and consequently the present status of Japanese physician trainees’ case summarization skills is unclear. Additionally, although the importance of summary statement practice to CR development within oral presentations and VPs has been identified [9, 13], its effectiveness has not been evaluated. This study used VPs to explore Japanese 1^st^ year trainee physicians’ (“junior residents”) styles of case summarization and the effectiveness of VPs on developing case summarization skill.

## Methods

We conducted a prospective study from October 2012 to May 2013 to explore clinical case summary statement style, and change in both the summary statement style and CR within the summary statement with progressive VP use. The study was approved by institutional review boards at the University of Pittsburgh and St. Luke’s International Hospital, and comparable governing boards at Teine Keijinkai Hospital, Mito Kyodo General Hospital, and Ebetsu City Hospital.

### Virtual patient platform

In conjunction with the Computer Science Department at the Tokyo University of Technology, we developed five VP modules using Adobe Flash© software. Each module was a computerized transformation of a clinical case previously experienced by a Japanese junior resident physician, and identified as having CR educational value for same-level trainees. Cases included adolescent pancreatitis, pediatric mycoplasma pneumoniae, duodenal ulcer perforation, pulmonary embolism, and acutely altered mental status. Incorporating a reference bank of symptoms, risk factors, and examination findings for relevant clinical conditions, modules focused on diagnostic and decisional thought processes to enable successful completion without requiring specialized knowledge of topics. The diagnostic portion included a brief history of present illness followed by interactive lists of additional history-taking and physical examination items; immediate feedback on the appropriateness of each item was provided as items were selected. Participants then completed a free-text summary statement (SS) with instruction to summarize the relevant portions of the symptom history, pertinent risk factors, and physical exam findings. An exemplar SS adjacent to the participant’s SS appeared on the next screen (see Additional file 1 for exemplars). Each module concluded with a linear pathway of interactive slides addressing further evaluation and management. Module content was provided in Japanese. Further details of the VP design have been described previously [7].

### Participants

Japanese post-graduate clinical training commences with a government mandated 2-year junior-residency including rotations in general surgery, internal medicine, pediatrics, and other “core” specialties pertaining to general practice. From September to October 2012, site coordinators at 4 Japanese post-graduate clinical training programs - designated Program A (15 residents), Program B (24 residents), Program C (10 residents) and Program D (5 residents) - invited all PGY1 level resident physicians (n = 54) to participate in the study. The Japanese academic calendar runs from April to March; hence the timing was early in the participants’ post-graduate training, but after adjustment to site-specific schedules and responsibilities. Participants at Program A had the opportunity to attend a Western style case discussion in English roughly once weekly, depending on their rotation schedules, which included an SS exercise. Participation was voluntary and informed consent was obtained from all subjects; participants received 1000 yen (roughly $9 U.S.) per module completed.

### Study design

Each potential participant was assigned a randomized sequence for completion of the 5 clinical cases as generated by the University of Pittsburgh Department of Biostatistics. Study site coordinators verbally oriented potential participants to the study. The data management server administrator at the Tokyo University of Technology then emailed each potential participant with reiterated study details and download access to the first VP module. In anticipation that monthly completion of five VP modules would be a reasonable real world expectation, reminders for subsequent assigned modules, including addresses for module download, were emailed at 6 day intervals. Participants were requested to complete each module before receiving access to the next module. For security reasons preventing online module download, USB memory sticks containing the modules in pre-randomized order were provided at Program B. Module responses were automatically transferred to the data management server and labeled with the participant’s ID number.

Upon commencing results analysis two weeks after completion of the planned study period, it was learned that some data had been lost in transmission from participant computers to the data management server and that some participants had not received emails from the server. These issues were believed due to server malfunction and poor compatibility between the server and commercial email accounts (e.g. Hotmail). Relevant participants were contacted and module access was extended by 3 months. Participants did not repeat any portions of the study. Data for 2 participants were added due to this modification.

This study was conceived as a pilot study and lacks a control arm of participants not assigned to VPS modules. Given the lack of prior data on summary statement styles and clinical reasoning, a power calculation could not accurately be performed to estimate a suitable population size for such a trial.

### Categorization of summary statement styles

Using thematic analysis, two investigators (BH, NK) independently reviewed all summary statements from the first VP module to identify emerging stylistic categories. Consensus was achieved through discussion. Independent review of all summary statements from the final VP module identified no new categories. Each investigator then labeled each summary statement into respective categories.

### Assessment of clinical reasoning from summary statements

Each VPS module contained information essential to diagnosis and management, items known as Key Features (KF) [21]. Clinical vignette-based problems targeting KFs have been previously validated for assessment of clinical reasoning [22, 23]; recommended question formats include write-in answers [24]. We developed a scoring rubric for the summary statement in each VP module. Key features of each clinical case were identified through input from the 5 clinical educator co-investigators and the JAMA textbook, *The Rational Clinical Examination* [25], where applicable. These co-investigators applied a score from 1 to 5 (least to most relevant to case) for each feature, and scores were averaged to create the final scoring rubric. (Copies of the rubric are available by contacting the lead author.)

For summary statements addressing multiple differential diagnoses, points were awarded for each KF supporting the correct diagnosis or refuting an alternative diagnosis. As brevity is essential to concise summary statements, 2 points were deducted for each element that did not contribute to the diagnosis (e.g. “negative Murphy’s sign” in describing physical exam findings for a child with mycoplasma infection).

Using methods validated by Bordage and colleagues, [16] the data coders evaluated semantic qualification by identifying whether or not each SS was semantically driven. Differences were resolved through discussion.

### Statistical analysis

Inter-rater agreements for both the SS style categorization and the SS CR scores were measured using Cohen’s kappa statistic.

Comparison of participants’ SS style and performance, respectively, between module #1 and each subsequent module, was performed using non-parametric tests where possible in consideration of the sample size. Pearson’s chi-square test was used to compare distributions of SS styles (Fisher’s exact test was not possible due to different participant numbers in the comparison groups). McNemar’s test was used for paired comparison of application of the modeled SS style versus other styles. Non-paired Wilcoxon Rank Sum test was used to compare SS CR performance as determined by the scoring rubric described above. Paired tests were not used due to the unavoidable variation in difficulty between VP cases. This variation was addressed by randomizing the VP case (i.e. module) sequence for each participant as described above, and then comparing performance of the participants collectively on respective modules. In addition, Chi-square and McNemar’s tests were used to compare collective and paired demonstration of semantically driven summarization, respectively, on modules #1 and #5.

Data were also stratified by study site to address the effect of individual program differences. Chi-square and Wilcoxon Rank Sum tests were used to compare SS style distribution and CR quality, respectively, on modules #1 and #5. All tests were 2-sided and the level of statistical significance was specified at 0.05. Stata Version 13.0 statistical software (StataCorp, College Station, TX) was used for all analyses.

## Results

Thirty-nine, 36, 35, 26, and 29 participants completed module 1, 2, 3, 4, and 5, respectively. The median self-reported duration to complete each module was 10–15 minutes (range: <10 min. to >20 min.). Of the 165 entered summary statements, 15 were eliminated due to paucity of content and 3 were transmitted in unintelligible character format, resulting in 147 summary statements included in the analysis.

### Change in summary statement style

**Fig. 1:**
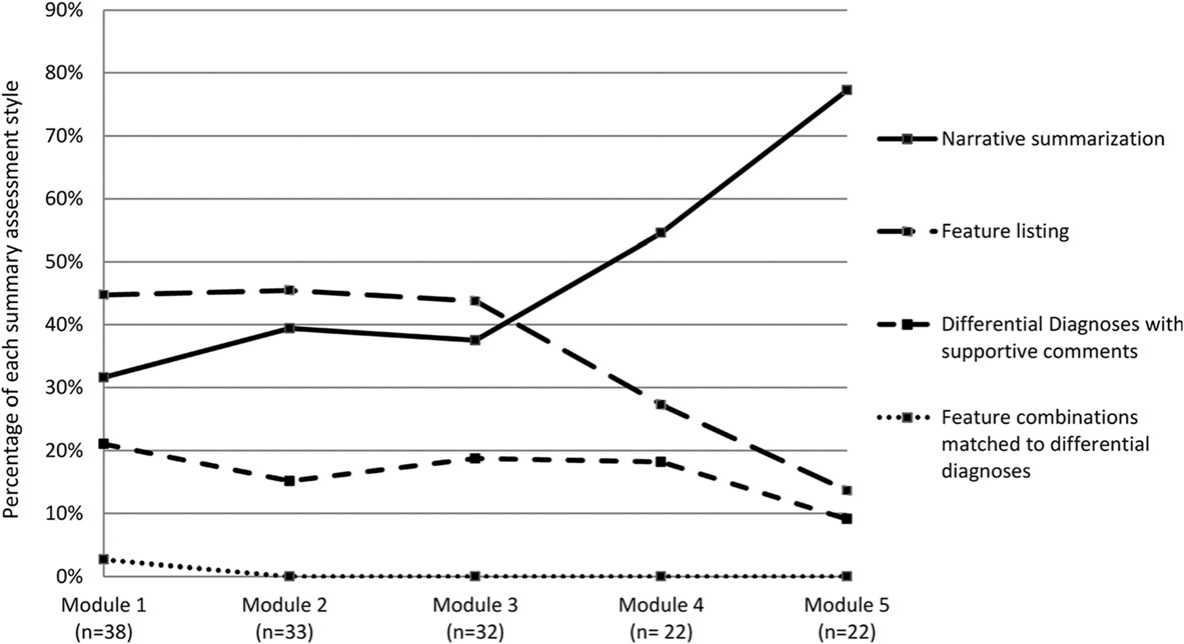
Change in percentage of summary assessment style over course of 5 VPs modules

Four SS styles were identified: numbered features matched to differential diagnoses (NMD), differential diagnoses with supportive comments (DD), feature listing (FL), and narrative summarization (NS) (modeled style) (Table 1). Inter-rater agreement was high, with a kappa of 0.841 (95 % CI 0.767-1.000). Figure 1 shows the distribution of respective styles for each module. From module #1 to #5, the demonstration of NMD, DD, and FL each decreased (2.6 – 0 %, 21.1 – 9.1 %, and 44.7 % – 13.6 %, respectively), while the demonstration of NS increased from 31.6 % to 77.3 %. Secondary to the shift from NMD, DD, and FL, to NS, significantly different style distributions were confirmed between module #1 and #5 (*p* = 0.008). Paired analysis of summarization styles restricted to participants completing both module 1 and respective subsequent modules (e.g. paired performance on modules #1 and #3 for participants who completed SS for both modules) demonstrated significant increase in narrative summarization from module #1 to modules #4 (*p* = 0.016) and #5 (*p* = 0.016), respectively.

**Table 1:** Identified summary statement styles with examples (translated from Japanese)

Feature combinations matched to differential diagnoses The participant selects and numbers information from the case, and then derives diagnostic hypotheses from combinations of this information. (Case of child with mycoplasma pneumonia infection and erythema multiforme) *#1 Fever (temperature 37.6 °C), #2 abdominal pain + abdominal tenderness, #3 Ileocecal lymphadenopathy on abdominal ultrasound, #4 rash, #5 respiratory rate 20 breaths per minute.* *#1、#2 = I think of acute enteritis. #3 finding, #4 = thinking about [causes of] erythema multiforme, Yersinia enteritis is very likely. #5 = I am thinking about mild tachypnea in addition to fever and abdominal pain.*
Differential diagnoses with supportive comments The participant generates several differential diagnoses and supports each one with brief isolated information from the case. (Case of elderly man found down, poorly responsive, and with evolving necrotizing fasciitis) *Hyperglycemia* ▪*History of diabetes mellitus without any prescribed medications.* ▪*Altered mental status present (disorientation)* ▪*Diabetic ketoacidosis due to hyperglycemia is possible.* *Hypothermia* ▪*Altered mental status occurring after being found in cold location.* *Carbon monoxide poisoning* ▪*In consideration of Sapporo [northern city] location, carbon monoxide poisoning caused by a heater cannot be excluded.*
Feature listing The participant lists information he/she thinks are important for diagnosis, but does not attempt to organize the information into a script. (Case of elderly man found down, poorly responsive, and with evolving necrotizing fasciitis) *81 y/o Man. Chief complaint: After falling with loss of consciousness, he is unable to speak.* *His grandson found him lying in prone position in bathroom and called for an ambulance.* ▪*No alcohol or containers nearby* ▪*Diabetes* ▪*Alcohol consumption yesterday unclear* ▪*No insulin* ▪*No oral medications* ▪*General appearance: confused, able to move all extremities on command* ▪*Vital signs: temp. 33.2 °C Respiratory rate 38 heart rate 82 blood pressure 122/67 SpO2 96 % (room air)* ▪*Pulse is normal but cyanosis is present* ▪*Cranial nerves grossly intact* ▪*Slight bilateral lower extremity pitting edema* ▪*No abnormality in sensation or strength* ▪*Deep tendon reflexes within normal limits* ▪*Babinski reflex within normal limits*
Narrative summarization The participant organizes important information as a narrative. (Case of child with manifestations of Mycoplasma pneumoniae infection including erythema multiforme) *9 y/o girl with history of febrile seizure and mycoplasma infection presents to the hospital with one day of continuous abdominal pain and temperature 38 °C. Yesterday, due to elevated inflammatory markers on blood work, she received outpatient intravenous fluid, but due to continued fever and abdominal pain, as well as decreased appetite, returned to the hospital. Regarding sick contacts, at her school there is an upper respiratory infection with predominant cough going around. On physical exam, excluding temperature 37.6 °C, vital signs are normal. On abdominal exam, there is a rash, no peritoneal sign, and generalized mild tenderness. Also, the rash is seen on her back and over entire lower extremities including soles of her feet.*

### Change in summary statement clinical reasoning

**Fig. 2:**
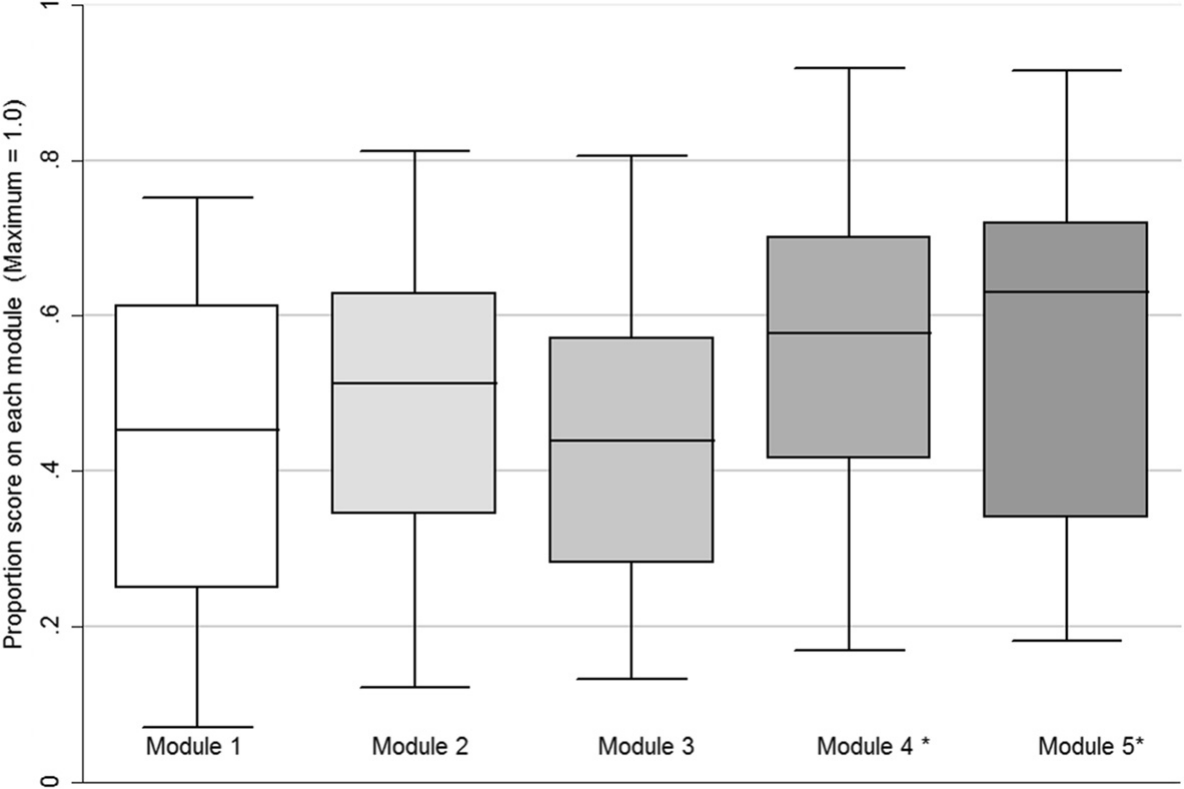
Clinical reasoning quality of summary assessments completed in VP modules. Legend: Scores determined by Key Feature rubric. *Compared to Module 1, improvement in performance achieved statistical significance on Module 4 (*p* = 0.040) and Module 5 (*p* = 0.021)

Our scoring rubric demonstrated very high inter-rater agreement with a kappa of 0.933 (95 % CI 0.897 - 0.969). Figure 2 contains a box plot of the average scores for each module. Significant improvement from baseline was observed for module #4 (*p* = 0.040) and module #5 (*p* = 0.021), respectively. Repeating comparison with scores restricted to the 22 participants completing summary statements for modules #1 and #4, and modules #1 and #5, respectively, revealed significant improvement in case summary statement CR from baseline for performance on module #4 (*p* = 0.036). While there was a trend toward improvement, summary statement CR improvement on module #5 failed to achieve significance (*p* = 0.060). It is anticipated that a larger sample size likely would have resulted in a lower *p* value.

Case summarizations were also confirmed to be more semantically driven in module #5 than #1, collectively comparing all summarizations from the respective modules (*p* = 0.003) and using paired analysis limited to the 22 participants completing both modules (*p* = 0.016). Table 2 contains two SS extractions from the VP case of altered mental status to demonstrate the concept of semantic driving. Both report similar data, but the semantically driven case summarization contains increased transformation of the patient’s history into meaningful abstractions (e.g. “duration of altered mental status unclear”) and articulation of the relationships between information (e.g. “day after drinking alcohol, he was found with altered mental status”).

**Table 2:** Examples of symptom versus semantically driven summary statements. Both extractions describe the case of altered mental status

Symptom driven case summarization	Semantically driven case summarization
*81 year-old man with history of diabetes. Today at approximately 4:30 pm, the patient’s grandson found him lying prostrate in his bathroom. His upper body was unclothed and he was wearing underwear. His underwear was stained with urine. The patient responded when called to. He was emergently transferred to our hospital. There is a history of alcohol use the night before admission.*	*81 year old man with a history of diabetes, but this is untreated. The day after drinking alcohol, he was found with altered mental status. The duration of altered mental status is unclear, but it persists at time of evaluation.*

### Comparison of participating programs

Evaluation of SS style distribution among participating programs found Program D (5/38 participants) to be significantly different from remaining programs for module #1 (*p* = 0.002), having more NMD and DD, and less NS, but no differences were appreciated for module #5. Regarding summary statement CR quality, no significant differences were appreciated among participating programs on module #1. However, Program B (6/24 participants) performed significantly worse than other programs on module #5 (*p* = 0.015), collectively performing significantly worse on their final module than on the previous modules that they completed. Two summary statements lacked physical exam items; 2 other summary statements demonstrated anchoring on incorrect diagnoses based on unrelated medical history.

Participants from Program A, where the regular curriculum afforded opportunity for SS practice, did not significantly differ from other participants in SS style (*p* = 0.527 and 0.423 for Modules #1 and #5, respectively) or CR quality (*p* = 0.433 and 0.171 for Modules #1 and #5, respectively).

## Discussion

This pilot study examined the clinical case summary statement, qualitatively with regard to style and quantitatively in measuring clinical reasoning. To our knowledge, this is the first investigation of the SS from these perspectives. The VP platform offers additional study result implications to medical education systems, in Japan and beyond, which lack a robust CR educational component and would benefit from a modality that efficiently helps develop this important skill.

### Summary statement style

In our exploration of SS style, 4 distinct categories emerged. We described the modeled style as “narrative summarization” to distinguish it from the remaining styles, though to our knowledge it has not previously received this label. This form of summarization is a hallmark of Western clinical training, intrinsically suited to the problem representation and key feature organization that enhance knowledge encapsulation [8, 9]. In this manner, summarization articulates illness scripts that can be applied to future clinical practice. Importantly, narratives are a primary means of human learning spanning all cultures [26, 27]. As participants received neither positive nor negative feedback on their summary statements, nor did they receive incentives external to the study, we hypothesize that the statistically significant adoption of the narrative SS in part reflects their intrinsic comfort with this style. In terms of improving clinical reasoning, other identified SS styles identified in our study, though potentially serving as didactic exercises and assisting diagnosis in some cases, may be less effective in developing problem representation and illness script formation.

The implications of our findings are especially relevant to our participants’ level of training as it is during the PGY1 year that Japanese physician trainees begin to practice extensively with patients, thus representing the peak period for clinical knowledge encapsulation and illness script development [20]. Regarding the potential influence of different teaching styles amongst study sites, only Program D participants demonstrated significantly different SS styles from the remaining programs. The small n (5/38 participants), including the isolated participant who numbered and matched case data to differential diagnoses, precludes conclusions about teaching methodology unique to Program D.

### Summary statement clinical reasoning

Clinical reasoning quality in summary statements significantly improved over the course of the study when assessed by both Key Features and semantic driving. These data are consistent with previously published data suggesting CR enhancement during the VP cases, including pre-post change in Diagnostic Thinking Inventory score (*p* = 0.07) [7].

Summary statement quality may reflect the trainee’s articulation of CR, distinct from SS style, as well as CR, itself. From our data, arguments can be made for the influence of both factors. Articulation of CR appears more influential when the SS is viewed stylistically. Similar to their adoption of the modeled narrative summarization, in demonstrating semantically driven SS and improved KF rubric scores, participants may have used the SS exemplar to better express knowledge and reasoning they already possessed. In contrast, when the SS is interpreted through the lens of CR, the significant increase in semantically driven SS identifies improvement in reasoning. This finding is intertwined with the different observed SS styles, which may be interpreted as advancement from Bordage’s dispersed (Table 1, style #1) to elaborated (Table 1, style #4) stages of diagnostic thinking [28]. Regardless of which was more influential to the observed results, both CR and CR articulation are important skills which appear to increase with VP utilization.

Inconsistent participant effort may have influenced the summary statement CR quality results. Participants were informed beforehand that module results were de-identified and unrelated to formal evaluation of residency performance. Given the extensive clinical responsibilities competing for their time [6], it is likely that effort varied among participants and among modules for individual participants. For example, limited effort appears contributory to the decreased performance of Program B participants on Module #5 and may have influenced the failure to achieve overall statistical significance (*p* = 0.060) compared to baseline. Despite this barrier, improvement achieved statistical significance after 4 modules, suggesting that participants valued the exercise.

### Strengths and limitations

This study has strengths that enhance extrapolation of the results to real world implementation of these VP cases. The training experience for Japanese resident physicians includes increased clinical demands and decreased educational opportunities compared to Western resident physicians [6, 29]. The study, including VP design and rollout, was designed for these real-world conditions and proved to be successful.

This study has important limitations. First, outcome measures for CR quality included adaptation of the previously validated KF question format to summary statement analysis. Content validation was achieved amongst five clinician educators and concurrent validity is supported by consistency with other outcome measure data mentioned above, but more rigorous construct validation studies are needed to improve confidence in this methodology. Second, email correspondence between server administrator and participants was unreliable, and malfunction in data transfer to the server caused data loss and extension of study period. Third, there was no control arm of participants assessed for SS or CR but not completing VPS modules. While a causal effect of the intervention on SS outcome measures is supported by the comparable results between Program A and the remaining programs, it is possible that outcomes were influenced by other experiences in participants’ training. Finally, in exploring the SS styles, we adopted the dominant paradigm based on cognitive psychology. Approaching the analysis using an alternative conceptual framework, for example cognitive load theory or narrative medicine, may yield different understanding and categorization of the participants’ summary statements.

## Conclusions

In our prospective study involving Japanese resident physicians, we identified four styles of summary statements, observing both the adoption of the narrative style and improvement in the clinical reasoning quality of summary statements during a series of VP cases. For this physician trainee population facing high clinical work demands and lack of structured guidance from clinician educators, this educational supplement offers an efficient means to develop this important skill. The encouraging results identified here warrant repeating this study on a larger scale with a control population, as well as establishing an association with meaningful clinical outcomes.

## Additional file

Additional file 1:
**Exemplar summary statements of clinical cases used for this study.** Japanese translations were used in the study. (DOC 30 kb)
