# Supplementary material for: Virtual patients to explore and develop clinical case summary statement skills amongst Japanese resident physicians: a mixed methods study
Source: BMC Med Educ. 2016 Feb 1;16:39. doi: 10.1186/s12909-016-0571-y (PMC4736711; doi:10.1186/s12909-016-0571-y)
Supplement: Additional file 1: — Exemplar summary statements of clinical cases used for this study. Japanese translations were used in the study. (DOC 30 kb) [file 12909_2016_571_MOESM1_ESM.doc]

**Additional file 1: Exemplar summary statements of clinical cases used for this study.**

**Japanese translations were used in the study.**

Case 1: Adolescent boy with acute appendicitis

*16 year-old boy presents with 3 days of abdominal pain that has gradually changed from diffuse mild pain to focal RLQ more severe pain. He has loss of appetite and subjective fever.*

*Exam reveals a boy who is mildly ill-appearing, but also joking. There is mildly elevated temperature and isolated tenderness at McBurney’s point. There is no guarding or rebound tenderness.*

*Except for the time course, which is slightly long, this presentation is very consistent with appendicitis.*

Case 2: Child with multiple signs and symptoms of Mycoplasma pneumoniae infection, most specifically erythema multiforme.

*Previously healthy 9 y/o girl with respiratory infection two weeks prior presents with a three day progression of fever, intermittent abdominal pain, soft stool, and oligoarticular joint pain.*

*The pt appears ill but maintains clear mentation. She has a low grade fever but vitals are otherwise WNL.*

*Physical exam is remarkable for mildly injected throat, diffuse abdominal tenderness with hyperactive bowels sounds, a slightly swollen ankle, and target lesions over the abdomen, back and bilateral lower extremities including the soles.*

Case 3: Elderly patient found down with altered mental status and isolated severe tachypnea, subsequently confirmed secondary to evolving necrotizing fasciitis.

*81 y/o man with diabetes is found down, lethargic, hypothermic, and tachypneic near his bathtub. He was reportedly in his USOH until the evening before presentation when he went out drinking. Estimated time down is about 10-18 hours. He reportedly does not take any meds, including insulin.*

*On exam in ER, vitals show mild hypothermia and severe tachypnea, but BP, HR, and SPO2 are WNL. He is confused but cranial nerves are intact and he can move all extremities. There is mild bilateral LE edema, but everything else is unremarkable, including the pulmonary exam.*

Case 4: Middle-aged man with acutely perforated duodenum secondary to H. pylori mediated ulceration.

*A 41 y/o man with history notable for CAD and past gastric ulcer presents with sudden severe epigastric pain in the setting of about two weeks of intermittent epigastric pain occurring in the middle of the night and resolving by the afternoon.*

*On presentation, he is mildly diaphoretic and with labored breathing, but speaking normally. Vital signs are remarkable only for tachypnea. Abdominal exam reveals guarding and rigidity and also exquisite tenderness of the epigastrium and RUQ.*

*In consideration of the HPI and h/o prior gastric ulcer, the most likely explanation for this patient’s peritoneal signs is a perforated ulcer, probably in the duodenum.*

Case 5: Elderly man with massive acute pulmonary embolism likely associated with his multiple myeloma

*86 year old man with a past medical history notable for multiple myeloma but no prior cardiopulmonary disease collapsed at home after several days of progressive malaise and vague epigastric discomfort. No melena or hematochezia.*

*On presentation, he is in respiratory distress and tachycardic, but able to converse normally. Temperature and BP are WNL. His lungs are clear, heart sounds are regular without murmur and there is mild epigastric tenderness. There appears to be jugular venous distension. Stool guaiac is negative.*
